# Variation of Photosynthesis, Fatty Acid Composition, ATPase and Acid Phosphatase Activities, and Anatomical Structure of Two Tea (*Camellia sinensis* (L.) O. Kuntze) Cultivars in Response to Fluoride

**DOI:** 10.1155/2013/109367

**Published:** 2013-08-19

**Authors:** L. X. Wang, J. H. Tang, B. Xiao, Y. J. Yang, J. Liu

**Affiliations:** ^1^College of Horticulture, Northwest A&F University, Yangling, Shaanxi 712100, China; ^2^College of Life Science and Technology, Yangtze Normal University, Fuling, Chongqing 408000, China; ^3^Tea Research Institute, Chinese Academy of Agricultural Sciences, Hangzhou, Zhejing 310013, China

## Abstract

The changes of photosynthetic parameters, water use efficiency (WUE), fatty acid composition, chlorophyll (Chl) content, malondialdehyde (MDA) content, ATPase and acid phosphatase activities, fluoride (F) content, and leaf anatomical structure of two tea cultivars, “Pingyangtezao” (PY) and “Fudingdabai” (FD), after F treatments were investigated. The results show that net photosynthetic rate (*P*_*n*_), stomatal conductance (*g*_*s*_), and transpiration rate (*E*) significantly decreased in both cultivars after 0.3 mM F treatment, but FD had higher *P*_*n*_, *g*_*s*_, and WUE and lower *E* than PY. Chl content in PY significantly decreased after 0.2 and 0.3 mM F treatments, while no significant changes were observed in FD. The proportions of shorter chain and saturated fatty acids increased and those of longer chain and unsaturated fatty acids decreased in both cultivars under F treatments. The contents of MDA increased after F treatments but were higher in PY than in FD. In addition, F treatments decreased the activities of ATPase and acid phosphatase and increased F content in both cultivars; however, compared with PY, FD showed higher enzymatic activities and lower F content in roots and leaves. Leaf anatomical structure in FD indicated that cells in leaf midrib region were less injured by F than in PY.

## 1. Introduction

F is naturally present in sediments and soils, with a concentration range from 150 to 400 mg kg^−1^ and generally in forms of various insoluble compounds with elements such as calcium, aluminum, and silicon [1]. F is released into water, air, and soil through natural weathering and human activities, such as coal combustion and discharges of industrial water and industrial waste [2, 3]. Phosphate fertilizer and F-containing pesticides are additional sources releasing F into the environment [4, 5]. Trace amount of F is beneficial for the growth of teeth and bones in mammals, with its recommended upper limit at 1.5 mg kg^−1^, but excessive F intake has adverse effects [6, 7]. F-containing foods, drinking water, and especially tea are the major sources of F accumulation in humans [8, 9].

Tea as the most popular beverage crop in China can selectively absorb F from soil and thus results in higher F concentrations in tea leaves, compared with other plants including pine (*Pinus banksiana*), subterranean clover (*Trifolium subterranean*), cocksfoot (*Dactylis glomerata*), scotch thistle (*Onopordum acanthium*), and *Salicornia brachiate* [10, 11]. Some studies have investigated the effects of F on the physiological metabolism of tea plants, such as toxic symptoms [12], growth [13], photosynthesis [14], respiration [15], and carbohydrate metabolism [16]. However, few studies have investigated varietal difference of tea plants on tolerance to F.

 Previous studies showed that the concentrations of F in tea plants are significantly different from variety to variety, even for those living in the same natural environment [17–19]. Because of the long-term allogamy, tea plants might have a wide range of genetic backgrounds that affect the absorption and accumulation of F [20, 21]. In this study, two tea varieties were selected and investigated to further clarify the varietal tolerance and physiological response of tea plants to F. The results may help screen the low F variety for tea planting.

## 2. Materials and Methods

### 2.1. Plant Materials and Growth Conditions

One-year-old tea plants derived from rooted cuttings of two cultivars of *Camellia sinensis*, “Pingyangtezao” (PY) and “Fudingdabai” (FD) were used in this study. Both PY and FD are traditional cultivars possessing good agronomic characters, and were provided by Tea Experimental Station of Northwest A&F University. The plants were grown in black plastic containers (37 × 28 × 8 cm) each containing 1,000 mL of one-fifth strength Hoagland solution [22] for a week, and then transferred to half-strength Hoagland solution. The containers were placed in a growth chamber set at 70% relative humidity, 25/20°C (day/night), and a 12 h photoperiod with photon irradiance of 200 *μ*mol m^−2^ s^−1^ [23]. The solutions were aerated continuously and replaced weekly. Three containers, each containing 35 plants, were used for each treatment. Ammonium fluoride of 0, 0.1, 0.2, and 0.3 mM were added separately to the solutions. After incubation for 1, 3 or 14 days, the plants were collected for the following physiological analyses.

### 2.2. Photosynthetic Capacity

A *Li-6400* portable photosynthesis system (*Li-Cor*, Nebraska, USA) was used to measure photosynthetic capacity of the third leaves after F treatments. Measurements were carried out from 09:00 to 11:30 in the growth chamber under the following conditions: photon flux density of 500 *μ*mol m^−2^ s^−1^, leaf temperature of 30°C, relative humidity of 60%, and CO_2_ concentration of 395 *μ*mol mol^−1^.

### 2.3. Fatty Acid Composition

Total lipids were extracted by using a method of Folch et al. [24] with some modification. Fresh leaves (2 g) were ground in 50 mL of cold isopropanol and mixed thoroughly. After filtration, the residue was extracted for 30 min in 50 mL of chloroform/methanol (2 : 1, v/v) and refiltered. The filtrate from the first fraction was evaporated in vacuum to remove isopropanol and then dissolved in 30 mL of chloroform-methanol (2 : 1, v/v). The two collected organic phases were mixed, added with 10 mL of 0.2 M sodium phosphate buffer, and vortexed for 10 min. The mixture was extracted and dried under nitrogen; the residue was dissolved in hexane and transesterified with 0.5 M sodium methoxide for 20 min at 50°C following the method of Zwiazek and Shay [25]. The reaction was stopped by the addition of glacial acetic acid and water. Known amounts of n-octacosane were also added as an internal standard before transesterification. The hexane phase containing the fatty acid methyl esters was collected for quantification on a* 6890N* gas chromatograph (*Aligent*, Wilmington, USA), and the parameters were set partially according to Guo et al. [26]. Specifically, 2 *μ*L of sample was injected with hydrogen as the carrier gas at a flow rate of 2 mL min^−1^ and injection temperature of 250°C. The column temperature was initially set at 50°C for 6 min and then increased at 12°C min^−1^ to 170°C and held for 25 min and finally decreased at 4°C min^−1^ to 24°C and held for 30 min. Flame ionization detector (FID) temperature was 250°C. The flow rates of air and nitrogen makeup gas were 300 mL min^−1^ and 30 mL min^−1^, respectively.

### 2.4. Leaf Chl Content

About 0.1 g of leaf tissues were ground in a mortar and pestle with 10 mL of 80% chilled acetone. The homogenate was centrifuged at 1500 ×g for 5 min. The resulting supernatant was collected and mixed with 10 mL of 80% acetone and its absorbance at 663 nm and 645 nm was measured on a* UV-1800* spectrophotometer (*Shimadzu*, Tokyo, Japan). Chl content was calculated according to the method by Knudson et al. [27].

### 2.5. Leaf MDA Content

Fresh leaf or root tissues (0.2 g) were ground in liquid nitrogen in a mortar; then 2 mL of phosphate buffer (pH 7.8) was added and the homogenate was transferred into a 25 mL test tube. The mortar was rinsed twice with 0.05 M phosphate buffer (pH 7.8) and the washed buffer solution was added to the test tube. After addition of 5 mL of 55 mM thiobarbituric acid, the tube was shaken. The tube was heated in a water bath at 100°C for 10 min. Then after cooling, the tube was centrifuged and the absorbance of the supernatant at 532, 600, and 450 nm was measured on the *UV1800* spectrophotometer according to the method of Wilbur et al. [28].

### 2.6. ATPase Activity

Fresh root tissues (0.5 g) were ground in a chilled mortar with 5% (w/v) polyvinylpyrrolidone and then homogenized with 1.2 mL of 100 mM potassium phosphate buffer (pH 7.0) containing 1 mM EDTA and 1% Triton X-100. The homogenate was centrifuged at 13 000 ×g and 4°C for 20 min, and the supernatant was used for ATPase and acid phosphatase assays. 

 ATPase hydrolysis assay was performed as described by Kim and Weber [29]. The enzyme extract (1 mL) was mixed with 4 mL of a reaction mixture containing 50 mM Tris HCl (pH 7.2), 0.4 M sucrose, 3.0 mM ATP, and 3.0 mM MgCl_2_ and incubated at 38°C for 30 min. The reaction was terminated by adding 0.4 mL of 30% trichloroacetic acid. ATPase activity was determined by measuring the release of inorganic phosphate [30].

### 2.7. Acid Phosphatase Activity

To initiate the phosphatase reaction, 1 mL of enzyme extract was added to 3 mL of a mixture containing 50 mM sodium acetate buffer (pH 5.0), 3 mM disodium p-nitrophenyl phosphate, and 1 mM dithiothreitol. After incubation at 25°C for 15 min, the reaction was terminated by the addition of 1.0 mL of 0.4 M NaOH. The absorbance of the mixture was measured at 410 nm, with a reaction terminated at *t* = 0 serving as a control, in accordance with the method of Ciereszko et al. [31].

### 2.8. F Concentration

Leaf or root powder (0.2 g) was digested in 4 mL of concentrated nitric acid in an *MDS-8* microwave digestion device (*Sineo*, Shanghai, China) set at 120°C for 2 min and 180°C for 2 min. The digested liquid was transferred into a 25 mL volumetric flask and diluted to volume with deionized water. After filtration through Whatman no. 40 filter paper, the filtrate was used for ion detection with a *Dual Star* F ion selective electrode (*Thermo Fisher*, Swedesboro, USA).

### 2.9. Histological Observation

Newly expanded leaves were used for histological observation. Procedures used for obtaining ultrathin sections and section staining were described in detail by Foster [32]. Ultrathin sections stained by toluidine blue (0.1%) were observed with an *Axio Imager A1* light microscope (*Zeiss*, Shanghai, China).

### 2.10. Statistical Analyses

All measurements were performed four times, and the data were expressed as means ± standard deviations (SD). All collected data were subjected to analysis of variance using OriginPro 8.5.1. Significant level at *P* < 0.05 was calculated using Fisher's *F* test.

## 3. Results

### 3.1. Photosynthetic Parameters and WUE

**Figure 1:**
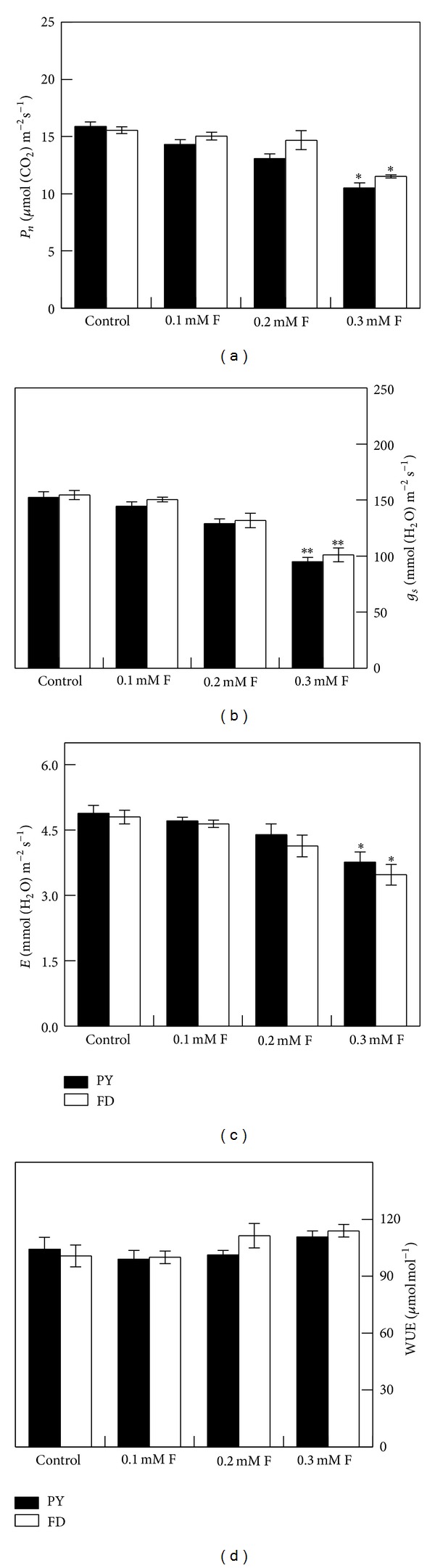
(a) Net photosynthetic rate (*P*_*n*_); (b) stomatal conductance (*g*_*s*_); (c) leaf transpiration (*E*); and (d) water use efficiency (WUE) after 2 weeks of treatments. Data are expressed as mean ± SD (*n* = 4). PY: “Pingyangtezao” tea cultivar; FD: “Fudingdabai” tea cultivar. * (**) indicates significant difference compared with the control at *P* < 0.05 (*P* < 0.01) according to Fisher's *F* test.

*P*_*n*_ (Figure 1(a)), *g*_*s*_ (Figure 1(b)), and *E* (Figure 1(c)) decreased after F treatments in both cultivars, but significant changes (*P* < 0.05) were only observed in 0.3 mM F treatment. WUE (Figure 1(d)) increased in both PY and FD after F treatments but was generally higher in FD than in PY.

### 3.2. Changes in Fatty Acid Composition in Leaves

The proportions of palmitic acid (16 : 0) and stearic acid (18 : 0) increased significantly in both cultivars after 0.3 mM F treatment (Table 1). The contents of these two acids were higher in PY than in FD. However, the proportions of arachic acid (20 : 0) decreased significantly in PY and FD after the same F treatment, but arachic acid contents in PY were higher than in FD. With respect to unsaturated fatty acids such as oleic acid (18 : 1) and linolenic acid (18 : 3), the responses of the two cultivars differed. Contents of linolenic acid (18 : 3) in FD significantly decreased after 0.2 mM and 0.3 mM treatments, while the content of oleic acid (18 : 1) in PY decreased only after 0.3 mM treatment.

### 3.3. Leaf Chl and MDA Contents

**Figure 2:**
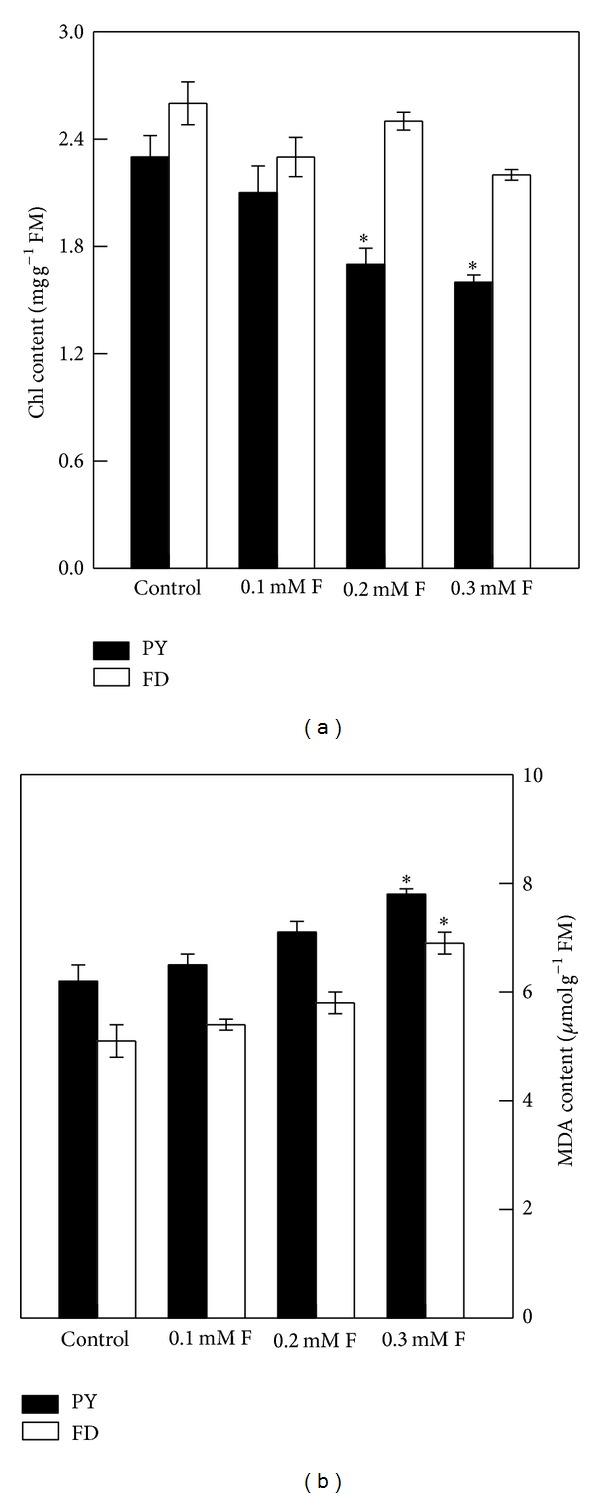
(a) Chlorophyll (Chl) content and (b) malondialdehyde (MDA) content in leaves of PY and FD seedlings after 2 weeks of treatment. Data are expressed as mean ± SD (*n* = 4). PY: “Pingyangtezao” tea cultivar; FD: “Fudingdabai” tea cultivar. * indicates significant difference compared with the control here at *P* < 0.05 according to Fisher's *F* test.

While Chl content in PY significantly decreased after 0.2 and 0.3 mM F treatments, no significant changes were observed in FD (Figure 2(a)). MDA contents in both PY and FD significantly increased after 0.3 mM treatment (Figure 2(b)). In the control plants, Chl content was higher in FD than in PY, whereas MDA content was lower in FD than in PY. 

### 3.4. ATPase and Acid Phosphatase Activities

**Figure 3:**
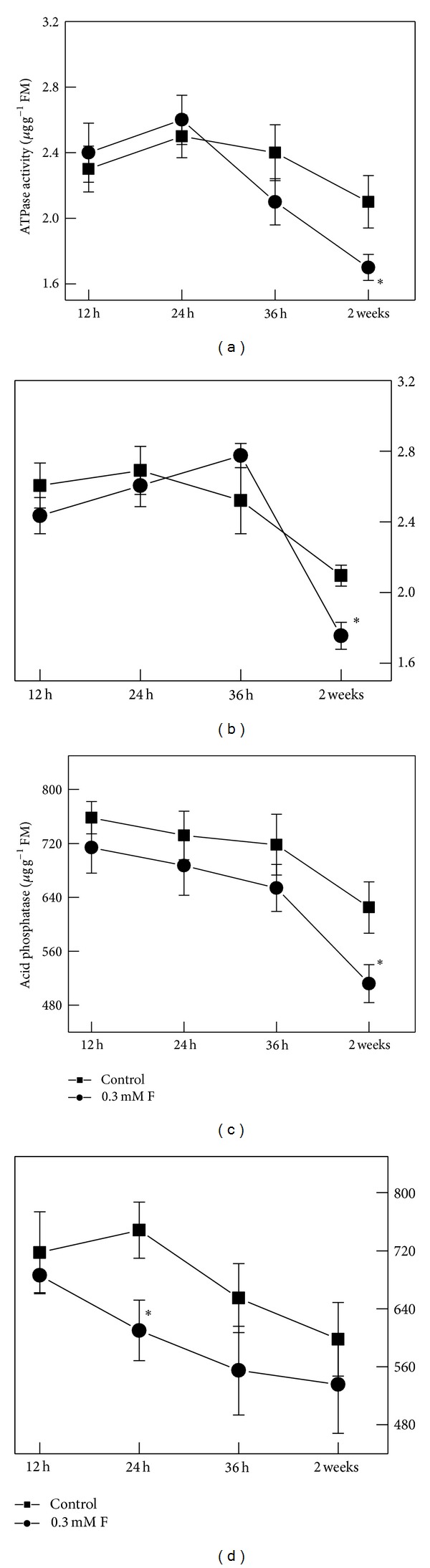
ATPase and acid phosphatase activities in roots of PY and FD seedlings after 12 h, 24 h, 36 h, and 2-week exposure to 0.3 mM fluoride (F). Data are expressed as means ± SD (*n* = 4). PY: “Pingyangtezao” tea cultivar; FD: “Fudingdabai” tea cultivar. (a) ATPase activity in PY; (b) ATPase activity in FD; (c) acid phosphatase activity in PY; (d) acid phosphatase activity in FD. * indicates significant difference compared with the control here at *P* < 0.05 according to Fisher's *F* test.

Temporal changes of ATPase and acid phosphatase activities were recorded in roots after F treatments. ATPase activity in PY increased after 24 h of 0.3 mM F treatment and then significantly decreased after 2 weeks (Figure 3(a)). A similar changing pattern was observed in FD: an increase after 36 h treatment and a decline after a longer treatment of 2 weeks (Figure 3(b)). Compared with the control plants, acid phosphatase activity significantly decreased in PY after 2-week treatment (Figure 3(c)) but significantly decreased in FD only after 24 h F treatment (Figure 3(d)).

### 3.5. Changes in F Concentration

Compared with the control plants, F concentrations in roots and leaves of both PY and FD increased significantly in all F treatments (Table 2). F concentrations in roots and leaves of PY were 1.2–1.6 times higher than in FD. A similar trend was observed in the control plants, with 17–25% increase in PY, compared with FD. 

### 3.6. Histological Observation

**Figure 4:**
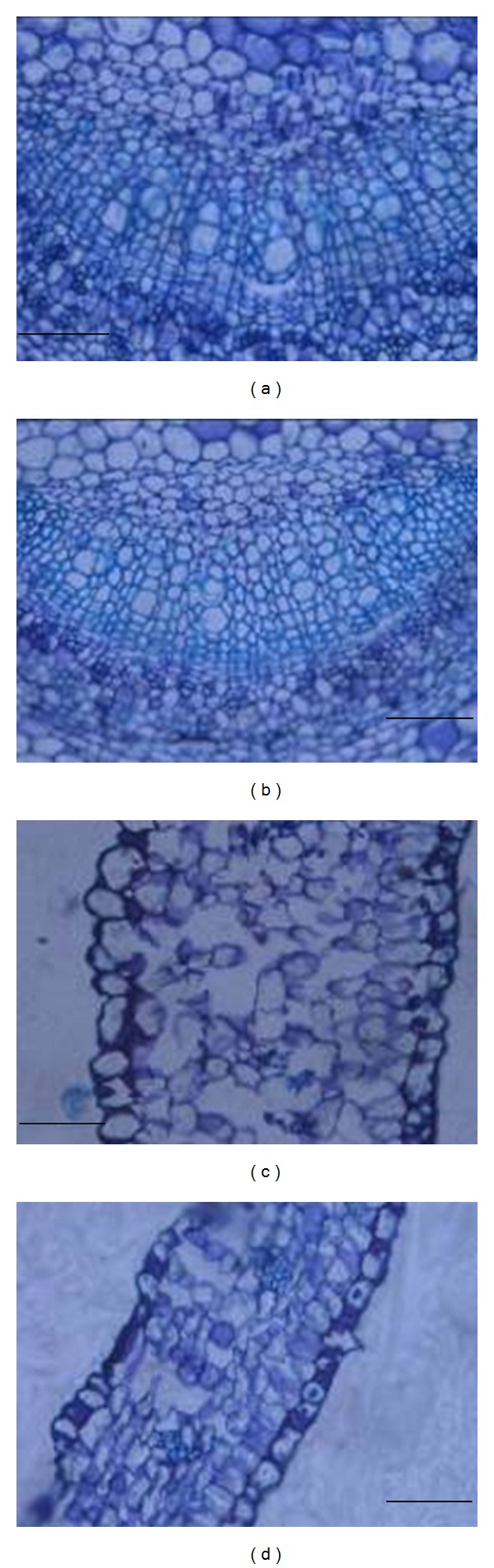
Comparison of root and leaf transections between PY and FD after 2 weeks of 0.3 mM fluoride (F) treatment. (a) and (c) represent midrib regions and leaf lamina, respectively, in PY; (b) and (d) represent midrib regions and leaf lamina, respectively, in FD. Scale bar = 100 *μ*m in (a) and (b); scale bar = 25 *μ*m in (c) and (d). PY: “Pingyangtezao” tea cultivar; FD: “Fudingdabai” tea cultivar.

F treatment induced more cell injury in the leaf midrib region of PY (Figure 4(a)) than in that of FD (Figure 4(b)), as evidenced by brown coloration upon histochemical staining. Compared with PY (Figure 4(c)), the arrangement of leaf lamina spongy mesophyll was more compact in FD (Figure 4(d)).

## 4. Discussion

Photosynthesis can be limited by two major processes: decrease of stomatal conductance and impairment of leaf photochemistry [33]. Another inhibitor of photosynthesis is the low plant water status, which can trigger stomatal closure [34]. Photosynthesis has been observed to decrease in *Picea *and* Pinus *after sodium fluoride treatment [34–36]. In our study, *P*_*n*_ (Figure 1(a)), *g*_*s*_ (Figure 1(b)), and *E* (Figure 1(c)) in both PY and FD significantly decreased after 0.3 mM F treatment, but the effects of F treatment were different between the two varieties. Under F treatments, FD had higher *P*_*n*_, *g*_*s*_, and WUE (Figure 1(d)) but lower *E* than PY. The decreased photosynthetic activity observed after F treatment may be the result of lowered *g*_*s*_. Several studies show that F treatment can inhibit hydraulic conductivity in root or shoot [14, 37], which might affect water balance in guard cells and induce stomatal closure. The decrease in *E* after F treatment observed in our study may also be ascribed to the lower *g*_*s*_. The higher *P*_*n*_ in FD compared with PY may be related to FD's higher WUE (Figure 1(d)) in leaf and lower *E* (Figure 1(c)). Maintenance of higher *P*_*n*_ and WUE and lower water loss by transpiration in FD than in PY indicates that FD is more tolerant to F.

Chl content is an important photosynthetic parameter, but it may not be directly correlated with photosynthesis. In this study, a higher leaf Chl content in FD control plants (Figure 2(a)) was not accompanied by a higher photosynthetic rate (Figure 1(a)) compared with PY control plants. This finding is consistent with observations in F-treated aspen seedlings [14]. While F treatment can reduce the leaf Chl content in cereals and aspen [14, 38], no obvious effect has been observed in *Salicornia* or *Chloris* [39, 40]. In our study, Chl content did not change significantly in FD with increasing F concentration but decreased significantly in PY. The effect of F treatment on Chl is not clear. Chl content was always higher in FD than in PY either with or without F treatment. These results might be explained by the anatomical structure of leaves. Mesophyll cells serve as storage areas for chlorophyll, and their concentration in laminae may reflect Chl content in leaves; we observed accordingly that the mesophyll cell layer in FD (Figure 4(d)) was tighter and thicker than in PY (Figure 4(c)). It may also be concluded that the effects of F treatment on Chl content differed between the two cultivars.

Lipids are esters of fatty acids and alcohols and comprise a large group of structurally distinct organic compounds that include fats, waxes, phospholipids, and glycolipids [41]. As major components of membrane lipids, fatty acids play an important role in maintaining normal physiological cell function under environmental stress, including temperature, salt, chemicals, ions, pressure, and oxidative stress [42]. F treatment can induce changes in lipid bilayer structure and alter the proportions of fatty acids [16]. Increase in palmitic acid content and decrease in linoleic acid content were observed in lipid fractions of F-treated* Sphagnum *and* Pinus *[16, 43]. In our study, the proportions of shorter chain and saturated fatty acids increased, while those of longer chain and unsaturated fatty acids decreased in both cultivars under F treatments (Table 2). These results appear to support the hypothesis of Simolal and Koskimies-Soininen (1980) [43] that F ions can inhibit the lengthening of fatty acid chains. The proportions of fatty acids in the two cultivars were different, but this might be a consequence of different lipid peroxidation levels. F can stimulate peroxidase and catalase activities in plants [16, 44, 45], inducing production of saturated fatty acids and MDA. In our study, MDA contents increased in both F-treated cultivars but were higher in PY than in FD plants (Figure 2(b)). Consistently, higher increases in levels of saturated fatty acids (i.e., palmitic acid and stearic acid) were observed in PY compared with FD. These differences may be ascribed to the more intensified peroxidation in PY than in FD under F treatment.

ATPase and acid phosphatase are widely distributed in different plant species and mainly localized in plasma membranes and vacuoles [46]. They are involved in the key metabolic functions of plants, such as growth, mineral nutrition, and transportation of stored metabolites [25]. F is known to be an inhibitor of activities of ATPase [47] and acid phosphatase [16, 48]. Though no changes in acid phosphatase activity were observed in plants exposed to F pollution by Yee Meiler (1975) [49], we observed decreases, as compared with controls, in ATPase and acid phosphatase activities in roots of both varieties after 2 weeks of 0.3 mM F treatment. A shorter treatment duration led to elevated ATPase activity in both varieties, but no increased acid phosphatase activity was recorded. This short-time activated enzyme activity may be due to that some other ions or organic anions combine with the enzyme to protect it from F inhibition.

In plants, F accumulation occurs mainly in mature leaves and may cause visual symptoms such as leaf tip and marginal chlorosis or necrosis [50]. The mechanism behind F toxic symptoms has not been fully elucidated. F-induced injury has been observed in mesophyll cell membranes and abundant deposits of lipid material have been found near plasmalemma, tonoplast, chloroplast, and mitochondrial membranes [16, 51], so cell membranes, are possible sites of F-induced damage. In this study, higher F concentrations (Table 2) were observed in PY leaves and roots, which may lead to more cell injury in the leaf midrib region than in FD (Figure 4).

In conclusion, F treatment can decrease photosynthesis and the activities of ATPase and acid phosphatase, change fatty acid composition, and cause anatomical structure injury in tea plants. The cultivar FD is more tolerant to F treatment, as it is characterized by better maintenance of photosynthesis and enzyme activity, better control of F absorption and membrane lipid degradation, and thus more efficient protection of metabolic processes and organic structure.

## Figures and Tables

**Table 1:** Fatty acid composition in leaves of PY and FD after 2 weeks of fluoride (F) treatments. Contents are given as percentage of total fatty acid weight. Data are the mean of four replicates. Minor fatty acids less than 1% of the total amount are not shown. Fatty acids are abbreviated as the number of carbon atoms followed by the number of double bonds in the fatty acid.

Fat acid	FD				PY			
	Control	0.1 mM F	0.2 mM F	0.3 mM F	Control	0.1 mM F	0.2 mM F	0.3 mM F
16 : 0	19.2	19.3	21.4	24.6*	20.3	21.1	22.4	25.8*
16 : 1	3.2	2.5	2.4	2.7	2.8	3.1	3.2	3.5
16 : 3	4.1	3.4	4.8	4.3	5.8	6.3	5.7	4.0
18 : 0	4.3	4.9	5.6	6.8*	7.1	7.5	8.4	9.2*
18 : 1	8.3	8.2	7.1	6.6	7.3	6.8	5.9	4.2*
18 : 2	11.5	13.5	12.5	13.7	15.6	13.9	14.4	15.8
18 : 3	41.1	40.6	38.4*	36.1*	32.6	31.8	31.9	30.2
20 : 0	7.4	6.1	5.2	3.8*	7.6	8.3	6.5	4.5*

*Indicates a significant difference compared with the control at *P* < 0.05 according to Fisher's *F* test.

PY: “Pingyangtezao” tea cultivar; FD: “Fudingdabai” tea cultivar.

**Table 2:** Fluoride (F) accumulation in roots and leaves of PY and FD after 2-week fluoride treatments (mg kg^−1^ DM). Data are expressed as means ± SD (*n* = 4).

Treatment	PY		FD	
	Root	Leaf	Root	Leaf
Control	0.5 ± 0.05	95 ± 4	0.4 ± 0.04	81 ± 3
0.1 mM F	2.2 ± 0.2***	167 ± 8***	1.4 ± 0.1***	124 ± 6***
0.2 mM F	3.6 ± 0.3***	210 ± 10***	2.1 ± 0.2***	177 ± 9***
0.3 mM F	5.6 ± 0.4***	269 ± 11***	3.4 ± 0.3***	226 ± 14***

***Indicates significant difference compared with the control at *P* < 0.001 according to Fisher's *F* test.

PY: “Pingyangtezao” tea cultivar; FD: “Fudingdabai” tea cultivar.
